# Differential Diagnosis of Corneal Manifestations in Hypovitaminosis and Retinitis Pigmentosa

**DOI:** 10.18502/jovr.v20.16803

**Published:** 2025-11-10

**Authors:** Beatrice Cesaro, Chiara Ancona, Mariantonia Ferrara, Giuseppe Nascimbeni, Francesco Semeraro, Vito Romano

**Affiliations:** Eye Unit, Department of Medical and Surgical Specialties, Radiological Sciences and Public Health, University of Brescia, Brescia, Italy

**Keywords:** Bariatric Surgery, Corneal Ulcer, Hypovitaminosis, Retinitis Pigmentosa

## Abstract

**Purpose:**

To report on the diagnostic and therapeutic approach in a patient with retinitis pigmentosa (RP), hypovitaminoses, and bilateral corneal involvement.

**Case Report:**

A 62-year-old woman, diagnosed with RP, presented with photophobia, sudden bilateral visual decline, and known long-term night blindness. Ophthalmic examination showed corneal perforation in the right eye, corneal ulcer in the left eye, and bilateral corneal hypoesthesia. She had undergone bariatric surgery and recently suspended vitamin supplementation. Blood tests revealed hypovitaminoses A, D, D3, and E. The patient underwent tectonic keratoplasty in the right eye and received topical therapy in the left eye, and then restarted vitamin supplementation as recommended by the nutritionist. Significant functional recovery was documented in both eyes at 1-, 3-, and 10-month follow-ups.

**Conclusion:**

The case highlights the importance of an integrative approach that includes comprehensive history taking, targeted laboratory work-up, as well as accurate differential diagnosis, especially given the potential multifactorality of ocular symptoms and signs.

##  INTRODUCTION

Hypovitaminosis is defined as a decreased vitamin serum level not satisfying the physiological needs. In developed countries, the main causes of hypovitaminosis are severely restricted diets, eating disorders, malabsorption, cystic fibrosis, chronic alcoholism, and bariatric surgery.^[1]^

A wide spectrum of clinical signs and symptoms can be associated with vitamin A deficiency (VAD). These include nyctalopia due to impaired rod function; conjunctival and corneal alterations such as xeroses, ulcerations, perforations;^[2]^ and a retinopathy characterized by subretinal hyperreflective deposits and alterations of the outer retinal layers.^[3]^ Differential diagnosis is especially important for nyctalopia, a typical symptom of inherited retinal dystrophies like retinitis pigmentosa (RP).^[4]^

We report a case of a patient with RP who had undergone bariatric surgery and presented with bilateral neurotrophic ulcers. The purpose of this case report is to highlight the importance of a holistic integrative approach to provide early diagnosis and intervention, particularly in complex patients. The patient was properly informed and, afterward, signed an informed consent for the publication of information and images.

##  CASE PRESENTATION

### Patient History and Presenting Symptoms

A 62-year-old woman reported a sudden bilateral decrease in vision and photophobia in addition to long-term nyctalopia (about 3 years). The patient had been followed up in another center for RP and had undergone bilateral cataract surgery. Upon admission to our unit, the patient did not exhibit any documentation regarding previous investigations performed to establish the diagnosis of RP. There was no history of trauma or contact lens wear. The patient had undergone bariatric surgery with biliopancreatic diversion, duodenal switch, and cholecystectomy in 2003 for severe obesity. Her past medical history included hospitalization for acute pancreatitis, microcytic anemia, hypocalcemia, and vascular encephalopathy in September 2022.

### Presenting Ocular Findings

**Figure 1:**
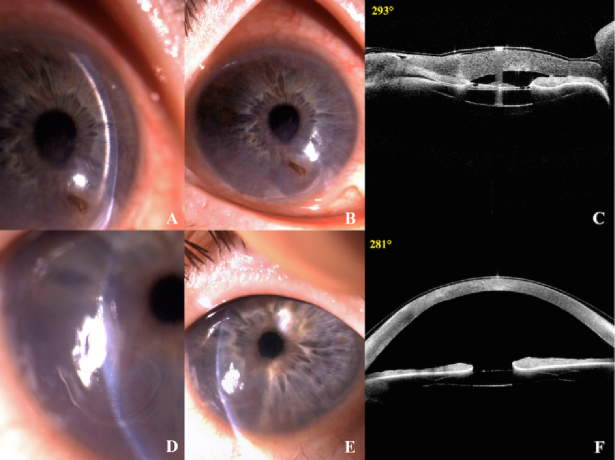
Slit-lamp photographs and AS-OCT of the right eye (A-B-C), showing a perforated corneal ulcer in the inferior nasal quadrant and the absence of AC. Slit-lamp photographs and AS-OCT of the left eye (D-E-F), showing a corneal ulcer in the inferior quadrant.

At presentation, best-corrected visual acuity (BCVA) was light perception in the right eye and 20/32 in the left eye. At slit-lamp examination, the right eye showed a perforated infero-nasal corneal ulcer measuring 3.2 × 3.0 mm and signs of active intraocular inflammation [Figure 1]. Additionally, the left eye was affected by an inferior corneal ulcer measuring 2.5 × 2.7 mm [Figure 1], and both eyes exhibited decreased inferior corneal sensitivity.

Anterior segment optical coherence tomography (AS-OCT) revealed an absent anterior chamber (AC) in the right eye, confirming the presence of perforation [Figure 1]. Fundus examination of the left eye showed a flat retina and typical RP findings, including bone-spicule-like pigmentation and arteriolar attenuation. Due to the condition of the anterior segment, it was not possible to perform ophthalmoscopy in the right eye. Therefore, a B-scan was performed to rule out any posterior complication that could potentially affect the management plan. Attention was paid to avoid exerting pressure on the perforated eye to prevent any complications. At B-scan, the vitreous cavity was unremarkable, and the retina was flat.

### Management

After corneal scrapings and placing of contact lenses, the patient was empirically treated with topical moxifloxacin 5 mg/mL and tobramycin 3 mg/mL six times a day in both eyes and oral doxycycline 100 mg once a day. The contact lens was placed on the perforated eye to act as a protective barrier and help stabilize the AC temporarily, whereas topical medications were applied to manage pain, inflammation, and infection. Scrapings were negative for infections, supporting the neurotrophic nature of the corneal ulcers. Due to the absence of systemic infection or concern for endophthalmitis, intravenous antibiotics were not administered.

Considering previous bariatric surgery, hypovitaminosis was suspected, and blood tests were performed. The latter revealed microcytic anemia, leukocytosis, and slightly elevated C-reactive protein level; however, the autoimmunity tests were otherwise negative, and renal, hepatic, and pancreatic function parameters were within normal limits. Serum vitamins analysis showed deficiencies of vitamins A (0.05 pg/mL, deficient level), D (12.0 pg/mL, severely deficient level), D3 (29 ng/mL, insufficient level), and E ( < 1 ug/mL, deficient level). Ocular findings were consistent with VAD. Moreover, based on the patient's medical history and laboratory tests, other potential differential diagnoses, such as infections, autoimmune and drug-induced diseases, zinc deficiency, and abetalipoproteinemia, were excluded.

The patient reported intermittent taking of multivitamin supplements for about 5 months prior to hospital admission. In addition, she suspended retinol and cholecalciferol supplements without medical consultation.

Right eye tectonic keratoplasty was performed 36 hours after the presentation. After inferior peritomy, a 5.0 mm peripheral manual trephination centered on the paralumbar corneal perforation was performed. This was followed by the release of the iridocorneal synechiae, suturing of a 5.0 mm donor corneal patch graft, reformation of the AC, affixation of the conjunctiva to the limbus with fibrin glue, and placement of a contact lens.

The therapy for the right eye was switched to topical netilmicin 3 mg/mL and dexamethasone 1 mg/mL, administered six times a day. In the left eye, prophylactic treatment with moxifloxacin 5 mg/mL and tobramycin 3 mg/mL six times a day was prescribed in combination with oral 100 mg doxycycline once a day. Vitamin A ophthalmic ointment and artificial tears were prescribed for both eyes. The patient was referred to a nutritionist and was treated with systemic retinol and tocopherol, vitamin D, a complete multivitamin complex, vitamin C, and iron supplementation.

### Follow-up

At 1-month follow-up, BCVA was counting fingers at 30 cm in the right eye and 20/25 in the left eye. The slit-lamp examination of the right eye showed the proper suture tension, proper placement and transparency of the corneal patch, and no sign of AC inflammation or infection. In the left eye, corneal epithelium was intact, with an inferior leucomatous opacity due to the previous corneal ulcer. The topical prophylactic therapy for the left eye was tapered to four times a day, and oral 100 mg doxycycline once a day was prolonged for 6 weeks.

**Figure 2:**
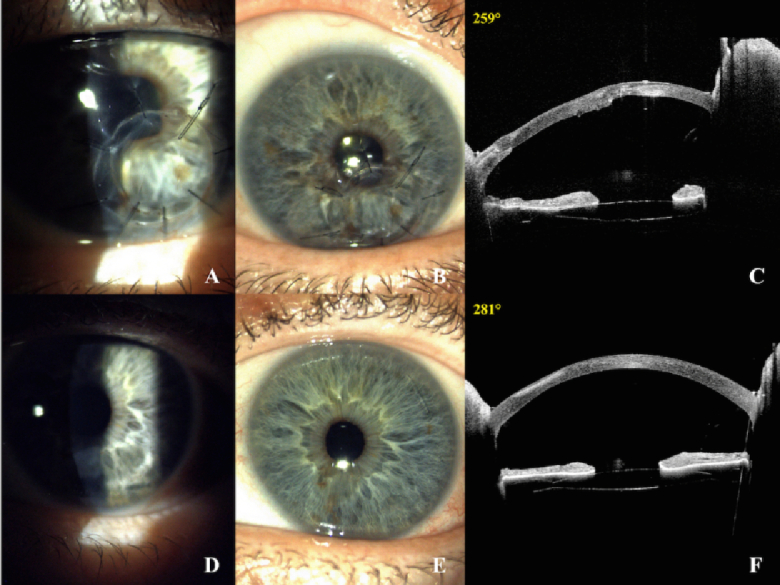
Slit-lamp photographs and AS-OCT of the right eye (A-B-C) at 10-month follow-up, showing proper suture tension as well as appropriate placement and transparency of the corneal patch. Slit-lamp photographs and SA-OCT of the left eye (D-E-F) at 10-month follow-up, showing leucomatous opacity in the inferior quadrant due to a previous corneal ulcer.

At 3-month follow-up, BCVA improved to 20/400 in the right eye and 20/20 in the left eye. Topical antibiotics were interrupted in the left eye, whereas topical therapy was continued in the right eye. Besides, vitamin A ophthalmic ointment at night and artificial tears four times a day in both eyes were continued.

At 10-month follow-up, BCVA was 20/25 in the right eye and 20/20 in the left eye. Fundus examination showed typical RP findings, including bone-spicule-like pigmentation and arteriolar attenuation in both eyes. The slit-lamp examination of both eyes is illustrated in Figure 2. A large keratoplasty was initially planned for the right eye, but it was not performed due to the good and satisfactory functional outcomes achieved [Figure 2].

##  DISCUSSION

Vitamin A is a fat-soluble vitamin obtained mainly from the diet.^[2]^ Subclinical VAD is defined as serum concentration between 0.35 and 0.70 μ mol/L (equivalent to 98 and 196 ng/mL, respectively), whereas serum levels < 0.35 μ mol/L are considered clinically relevant.^[5]^ In the eye, retinol is an important cofactor required for rhodopsin production. Consistently, VAD typically manifests with nyctalopia. Retinol also plays a role in ocular surface maintenance by controlling epithelial keratin expression and glycoprotein synthesis. Its deficiency leads to xerophthalmia due to loss of mucins and goblet cells in the conjunctival epithelium. The keratinizing squamous metaplasia of the conjunctiva forms Bitot's spots and a wide spectrum of corneal alterations, ranging from mild epitheliopathy to fulminant keratomalacia, ulcerations, and perforations.^[2]^ Retinol deficiency also correlates with the severity of inflammation in terms of pro-inflammatory cytokine levels and synthesis of the alpha-1 proteinase inhibitor.^[6]^

VAD is a known potential complication of intestinal and gastric bypass surgery as well as biliopancreatic surgery, with a reported 4-year incidence up to 69% following the biliopancreatic procedure.^[7]^ Fieldhouse et al reported recurrent corneal perforations in a woman with small bowel resection and subsequent malabsorption-related VAD.^[6]^ Interestingly, our patient presented with multiple hypovitaminoses (A, D, and E) due to bariatric surgery. She was also affected by RP, which could have been a potential confounding factor for the diagnosis of VAD due to some overlapping symptoms, in particular, nyctalopia. Retinal alterations, including retinal flecks, have also been associated with VAD; thus, other retinal dystrophies can be considered in the differential diagnosis.^[8,9]^ On the other hand, the differential diagnosis with RP is based on bilateral involvement, characteristic fundus alterations (such as bone spicules pigmentation, optic disc pallor, and arteriolar narrowing), and electroretinogram abnormalities.^[4]^ Additional distinguishing features between VAD and RP are the more common asymmetric involvement, the more rapid progression, and the concomitant corneal alterations in VAD.^[9]^ In both conditions, electrophysiological testing reveals dysfunction primarily affecting rod photoreceptors; however, VAD-induced alterations respond positively to vitamin A supplementation.^[4,9]^ Finally, a thorough family history is essential in any patient suspected for RP and can help with differential diagnosis. These aspects highlight the importance of history taking and patient-targeted blood tests for an appropriate work-up.

To our knowledge, this is the first case of a concomitant diagnosis of VAD and RP, a combination that could be a cause of delayed diagnosis of VAD. It is plausible that the patient sought care late due to the previous diagnosis of RP and the associated chronic night blindness. Early VAD diagnosis is also crucial because of the reversibility of the disease once appropriate vitamin A supplementation is started.^[10]^

In summary, this case report describes, for the first time, VAD-related ophthalmic signs in a patient with RP, and, more importantly, highlights the need to consider multifactorial causes in corneal perforations. One must consider VAD in the differential diagnosis of retinal dystrophies, even in the absence of its complete clinical spectrum. A holistic, integrative approach and a multidisciplinary team are essential to correctly manage these patients and provide early diagnosis and care.

##  Financial Support and Sponsorship

None.

##  Conflicts of Interest

None.
